# Endoleak after endovascular aortic repair and lumbar vertebral erosion

**DOI:** 10.1007/s10195-014-0329-4

**Published:** 2014-12-03

**Authors:** Antonio Bozzani, Vittorio Arici, Franco Ragni, Angelo Argenteri

**Affiliations:** Vascular and Endovascular Surgery, Foundation I.R.C.C.S. Policlinico San Matteo, P.le Golgi 19, 27100 Pavia, Italy

Dear Editor,

Mancini et al. [1] reported in their recent article an interesting clinical case on an 80-year-old man admitted to their hospital for severe low-back pain, lower limb motor impairment, and bilateral thigh pain. The patient had lumbar vertebral erosion due to an extensive infrarenal aortic false aneurysm secondary to an endovascular repair (EVAR). A CT scan performed one month after the EVAR did not show early procedural complications and the laboratotry findings performed during hospitalization were negative for infection.

Vertebral erosion secondary to an abdominal aortic aneurysm is rare. Generally, they are due to vascular prosthesis infections, chronic aneurysm rupture, or expansion. This is probably the first case secondary to EVAR. With growing numbers of interventional abdominal aortic procedures and increasing follow-up periods, complications of EVAR have become increasingly evident over time [2–5].

Via intraoperative fluoroscopy it seems that the endovascular prosthesis was a Trivascular Ovation (Trivascular Ovation Prime™, Santa Rosa, CA) comprising polymer-filled sealing rings that exert no chronic outward force. For this reason, the probability of a false aneurysm secondary to aortic neck rupture is low.

In our opinion, the cause of the false aneurysm formation is represented by the presence of an unrecognized endoleak or endotension that caused the tamponade aortic rupture.

Why was not an aortic CT scan repeated or a contrast-enhanced ultrasound (CEUS) performed?

## References

[1] Mancini F, Ascoli-Marchetti A, Garro L, Caterini R (2014). Aseptic lysis L2-L3 as complication of abdominal aortic aneurysm repair. J Orthopaed Traumatol.

[2] Arici V, Rossi M, Bozzani A, Moia A, Odero A (2012). Massive vertebral destruction associated with chronic rupture of infrarenal aortic aneurysm: case report and systematic review of the literature in the English language. Spine (Phila Pa 1976).

[3] Arici V, Quaretti P, Bozzani A, Moramarco LP, Rossi M, Carlino M (2014). Neck-targeted, stand-alone coiling for successful treatment of type 1° endoleak following endovascular repair. Vasc Endovasc Surg.

[4] Bozzani A, Odero A (2012). Late surgical conversion and inappropriate indications for TEVAR. Ann Thorac Surg.

[5] Pirrelli S, Arici V, Bozzani A, Odero A (2005). Aortic graft infections: treatment with arterial allograft. Transplant Proc.

